# Determination of Electrolytes and Trace Elements in Biological Samples from Patients with Altered Semen Parameters: a Correlational Analysis

**DOI:** 10.1007/s12011-024-04281-7

**Published:** 2024-06-26

**Authors:** Ahsanullah Unar, Hassan Imran Afridi, Asim Ali, Naveed Ali, Taiyaba Qureshi

**Affiliations:** 1 Department of Precision Medicine, University of Campania ‘L. Vanvitelli’, 80138 Naples, Italy; 2 Centre of Excellence in Analytical Chemistry, University of Sindh, Jamshoro, Pakistan; 3 Department of Biotechnology, COMSATS University Islamabad, Abbottabad Campus, Abbottabad, 22060 Pakistan; 4 Department of Political Science, University of Campania ‘L. Vanvitelli’, 81100 Caserta, Italy; 5 School of Computer Science, University of Science and Technology of China, Hefei, Anhui China

**Keywords:** Trace elements, Electrolytes, Infertility, Seminal plasma, Sperm

## Abstract

The effects of important nutrients such as calcium (Ca), copper (Cu), iron (Fe), magnesium (Mg), selenium (Se), and zinc (Zn) have been investigated in relation to male fertility due to their roles in proper spermatogenesis, sperm maturation, motility, and optimal sperm function. An imbalance between these elements has been associated with several pathologic conditions and male reproductive issues. The purpose of this study was to determine the essential trace and electrolytes elements, such as Ca, Cu, Fe, Mg, Se, and Zn, in human biological samples (blood, serum, and semen) from patients with male infertility. This study used correlational analysis to determine the potential associations between these elements and male fertility. Imbalances in these elements have been linked to various pathological conditions and male reproductive issues. One hundred eighty referent male adults and two hundred twenty-nine patients diagnosed with subtypes of infertility were included in the study, divided into two age groups. Acid digestion was controlled using a microwave oven, and the essential trace elements and electrolytes in the oxidized biological samples were determined using atomic absorption spectrometry. Certified reference materials of blood and serum were used to validate the accuracy of the methodology. The results showed that the concentrations of Ca, Cu, Fe, Mg, Se, and Zn in the blood, serum, and seminal plasma of male adults in all age groups were higher than those in patients with different infertility phenotypes. Essential element deficiency in all biological fluid samples may significantly negatively affect human reproductive health and lead to male infertility. Through a multidimensional approach, our study sought to unravel the intricate biochemical signatures associated with OAT, providing insights that may shape the landscape of diagnostic and therapeutic strategies for male reproductive health.

**Supplementary Information:**

The online version contains supplementary material available at 10.1007/s12011-024-04281-7.

## Introduction

Infertility is defined as the biological inability to conceive after at least one year of unprotected sexual activity [1]. 15 percent of couples suffer from infertility, with men responsible for thirty to fifty percent of instances [1]. Oligoasthenoteratozoospermia (OAT), characterized by low sperm count (Oligozoospermia (OZS)), poor sperm motility (Asthenozoospermia (AZS)), and abnormal sperm morphology (Teratozoospermia (TZ)), is a significant concern in male reproductive health [2] The intricate interplay of various factors contributing to OAT requires a comprehensive understanding of the biochemical and elemental milieu of the male reproductive system [3]. Electrolytes and trace elements, which are critical components in the regulation of cellular function and homeostasis, have been implicated in numerous physiological processes, including spermatogenesis and sperm function [4]. Male infertility has been associated with endocrine problems, genital tract infections [5], immunological deficiencies [6], aging, smoking, alcohol consumption [7], environmental factors (pesticides, toxins, radiation), and genetic diseases [8]. Despite recent advances in reproductive health, the cause of male infertility remains unknown. Several factors, including the environment, occupational exposures, socioeconomic status, and nutritional needs, might reduce sperm quality [9]. Scientists have examined the production and composition of sperm in the testes and adnexal glands to gain a better understanding of the male reproductive system [10]. Numerous studies have performed elemental analyses of the male reproductive system [10–12].

Multiple components of the human seminal plasma are essential for healthy sperm development and motility [10]. Seminal plasma contains considerable concentration of the trace elements Cu, Fe, Se, and Zn, along with the electrolytes Ca and Mg [10]. Owing to a deficiency in vital minerals, the quality of human sperm has decreased in recent years [10, 13].

Ca plays a crucial role in catalyzing the acrosome reaction in mammalian spermatozoa, which is essential for the sperm to penetrate the egg's outer layers. Additionally, there is convincing evidence that ca ions affect sperm motility. Ca influences various cellular processes within sperm, including flagellar beating and hyperactivation, both of which are critical for effective sperm motility and the ability to reach and fertilize the egg [14, 15]. In addition to affecting sperm development and motility, Mg is also an intracellular Ca antagonist [14, 15]. In seminal plasma and prostate secretions, the Mg- and Ca-dependent ATPase is associated with a pellet containing a few tiny granules and vesicles [14]. Mg is found in high concentration in the prostate gland, and after its released into the seminal fluid, the Mg concentration in the sperm decreases significantly [14, 16]. As a result, a substantial decline in sperm Mg levels may play a role in male reproductive issues [16].

The concentration of Zn in seminal plasma. is higher than in other organs [17]. It acts as a cofactor for a metal-fingered protein that binds to DNA. In addition to other DNA repair proteins, Cu/Zn superoxide dismutase also contains it [17]. Zn causes failure of sperm reproduction, hypogonadism, testicular volume reduction, low development of secondary sexual characteristics, and testicular development [1, 18].The lack of crucial antioxidant trace elements such as Se and Zn causes oxidative damage, resulting free radicals ‘quality and poor quality of patients’ spermatozoa, who smoke or engage in other types of addiction [19].

After Zn, Fe is part of many enzymes and metal-protein complexes and is the second most important metal [20]. Fe is essential for Deoxyribonucleic Acid (DNA) synthesis, oxygenation and reduction processes, electron transfer, and healthy cell growth and development; however, when excess Fe is present, the Fenton reaction results in dangerous free radicals [20]. High levels of Fe may increase lipid peroxidation in sperm, thereby reducing the percentage of normal sperm motility [20].

The metabolism of various metal proteins and enzymes, including chromosome oxidation enzymes, lysine oxidation enzymes, dopamine hydroxylase, and cytochrome oxidation enzymes, depends on the presence of Cu [21]. Superoxide dismutase enzymes (SOD) protect sperm cells from damaging reactions to reactive oxygen species and require Cu and Zn as cofactors [19, 20] When consumed in large quantities, Cu is toxic to many cells, including human sperm. The prostate is the main source of plasma Cu [21].

Se is a fundamental dietary component in male fertility preservation and other physiological processes [22]. Male infertility is thought to be caused by of 20–40 percent deficiency sperm production, [22]. The midpiece of spermatozoa contains glutathione peroxidases (GPxs), which are derived from selenoproteins and contain Se [22]. The consequences of Se deficiency on spermatozoa that have attracted the greatest attention include loss of sperm motility, midpiece fractures, and an increase in shape defects, particularly in the head [20, 22].

Increasing of industrialization, urbanization, and increase in population growth in Pakistan has led to significant biodiversity and environmental degradation [23, 24]. However, studies on the impact of toxic metals and other pollutants on male reproductive health in Pakistan are limited. The beneficial effects of essential trace elements and electrolytes on the male reproductive system are of scientific interest. However, few studies have been conducted on this topic due to a lack of information, awareness, and technical expertise [23].

The study involved the analysis of electrolytes and essential trace elements in biological samples from male subjects experiencing infertility. The aim was to understand the impact of these elements on sperm quality. The research explored the correlations between these factors and various findings from the seminograms of male infertility patients. This provided valuable insights for the diagnosis and treatment of male infertility.

## Materials and Methods

### Ethics Committee Approval

Before to sample collection, the study protocol was approved (Approval Number: NCEAC/2022/7/796) by the Ethics Committee of NCEAC, University of Sindh, Jamshoro, Pakistan. Written informed consent forms was obtained from the patients and healthy controls.

### Study Design and Pretreatment

The experimental group included 98 healthy adult male subjects [aged 15 to 30 years (*n* = 42) and 31 to 45 years (*n* = 56)] and patients with infertility based on spermiogram findings [aged 15 to 30 years (*n* = 116) and 31 to 45 years (*n* = 113)]. The infertility patients were categorized into the five subtypes: OZS [aged 15 to 30 years (*n* = 29) and 31 to 45 years (*n* = 27)], ASZ [aged 15–30 years (*n* = 23) and 31 to 45 years (*n* = 25)], oligoasthenozoospermia (OA) [aged 15 to 30 years (*n* = 21) and 31 to 45 years (*n* = 23)], OAT [aged 15 to 30 years (*n* = 24) and 31 to 45 years (*n* = 21)], and azoospermia (AZ) [aged 15 to 30 years (*n* = 19) and 31 to 45 years (*n* = 17)]. These participants were all selected from Hyderabad, Pakistan's urban area, based on individual requests. A questionnaire was presented to the participants to collect information about their consent, health, food preferences, and physical characteristics. Demographic data for all participants were obtained from a comprehensive database. The subjects had normal height and weight, no history of exposure to harmful chemicals, radiation, high temperatures, or physical injuries, and did not smoke or consume alcohol. At the time of selection, participants did not have any other physical conditions, such as hepatitis, diabetes mellitus, or infectious diseases. The study included both healthy male adults and patients with various types of infertility. Prior biological samples were collected; each participant underwent a thorough examination by an expert physician in the field.

### Sample Collection

Blood samples were collected by a registered male nurse using a Vacutainer syringe (Oxford, UK). Volunteers were asked to fast for the night before a sample of 10 ml blood was taken. The pathological results of samples collection and separation of serum from blood samples were performed according to standard protocols, which have been described in previous studies [25–30].

#### Semen Sample Collection

Semen samples were collected by masturbation from healthy adults’ male and five different types of infertile, aged between 16 and 45 years using sterile plastic vessels after a 72–120-h abstinence period and were sent to pathological laboratories in volumes of 2.0 mL within 25–30 min of collection. 2.0 mL were set aside for elemental analysis. Seminal plasma was separated using standard protocols of WHO laboratory manual for the examination and processing of human semen (Sixth edition, 2021).

### Microwave-Assisted Acid Digestion Method

All biological samples were duplicated using a microwave oven-based digestion procedure. Certified reference materials (CRMs) and biological samples were collected in six replicate samples. A total of 0.5 ml of each biological sample was treated with 1 ml of a freshly manufactured H_2_O_2_-HNO_3_ mixture (1:2, v/v). Ten milliliters of the mixture flask were heated in a microwave oven for 2–3 min at 950 MW power to finish the sample digestion. Before diluting with Milli-Q water up to 10 ml, the digested samples were placed at room temperature. These samples were analyzed for elements using atomic absorption spectroscopy (AAS). The same procedure was applied to prepare blank samples. To create a sensitive approach, CRMs of the blood and serum were used [23, 25, 26].

### Reagents and Glassware

Ultrapure water required for the processes was provided by Millipore (Milli-Q USA). Analytical chemicals such as hydrogen peroxide and nitric acid were purchased from E. Merck, Germany. Each sample was checked for contamination before used. Fluka Kamica (Buchs, Switzerland) used stock solutions containing 1000 ppm of Fe, Zn, Pb, and Cd that were fully verified. All standard solutions were serially diluted with 0.2 mol/L HNO_3_. CRMs of human blood and serum (Clinchek® Control, Lypholized, Germany, Munich, Recipe) were purchased to establish a sensitive method. For further investigation, standard and diluted solutions were made and kept in plastic bottles at 4–6 °C. Plastics and equipment were washed and rinsed with Milli-Q'd water after being submerged in 2 mol/L HNO_3_ for 24 h.

### Equipment Used

Matrices of biological samples were oxidized in a microwave oven at 900W with an acid mixture (HNO_3_ + H_2_O_2_ in a ratio 2:1). Essential trace element concentrations were determined using a double-beam Perkin-Elmer atomic absorption spectrometer model 700 (Perkin Elmer, Norwalk, CT, USA), which was equipped with a flame burner and graphite furnace HGA-400 (Perkin Elmer), a pyrocoated graphite tube with an integrated platform, and an AS-800 autosampler.

### Data Analyses

Excel X-stat and Minitab software were used for statistical analysis of the data. With a recovery rate of 98.7–99.6 percent for the verified element values, the method's validity was demonstrated to be the best (Supplementary Table S1). There was only a 1–2 percent variance in the mean values of each element, with a relative standard deviation (RSD) of 2 percent, and the samples took less than 5 min to digest entirely. The student’s t-test was employed to evaluate the statistical significance of the differences between numerical variables, in accordance with the distribution of the variables.

## Results

The biochemical parameters and clinical characteristics of all patients with infertility did not differ from those of healthy male adults of both ages. Most infertility patients have acceptable levels of Hb, percent, Hct, RBCs, WBCs, platelets, plasma glucose, fasting, whole blood HbA1c, serum LDL cholesterol, and triglycerides, comparable to adult male subjects, but infertile patients have lower blood HDL cholesterol levels. Serum testosterone levels in both categories of adult males were in the normal range in both categories of adult males, but 31–45-year-olds had the highest testosterone levels (*p* < 0.05). In all patients with infertility, testosterone levels decreased; however, in patients with azoospermia, testosterone levels were not observed. Semen analysis revealed that the semen volume, color, viscosity, and pH of adult male subjects and patients with all subtypes of infertility were consistent throughout time. Sperm concentration, sperm motility [progressive motile (%), non-progressive motile (%), and immotile (%)], and sperm morphology were reduced in patients with infertility (Supplementary Figure S1-S4). Comparison with data from male adults of different ages showed variations in elements in biological samples from infertile patients. Supplementary Table S2 shows the mean concentrations of essential trace elements and toxic elements in biological samples (e.g., serum, plasma, and blood) and their standard deviations.

**Fig. 1:**
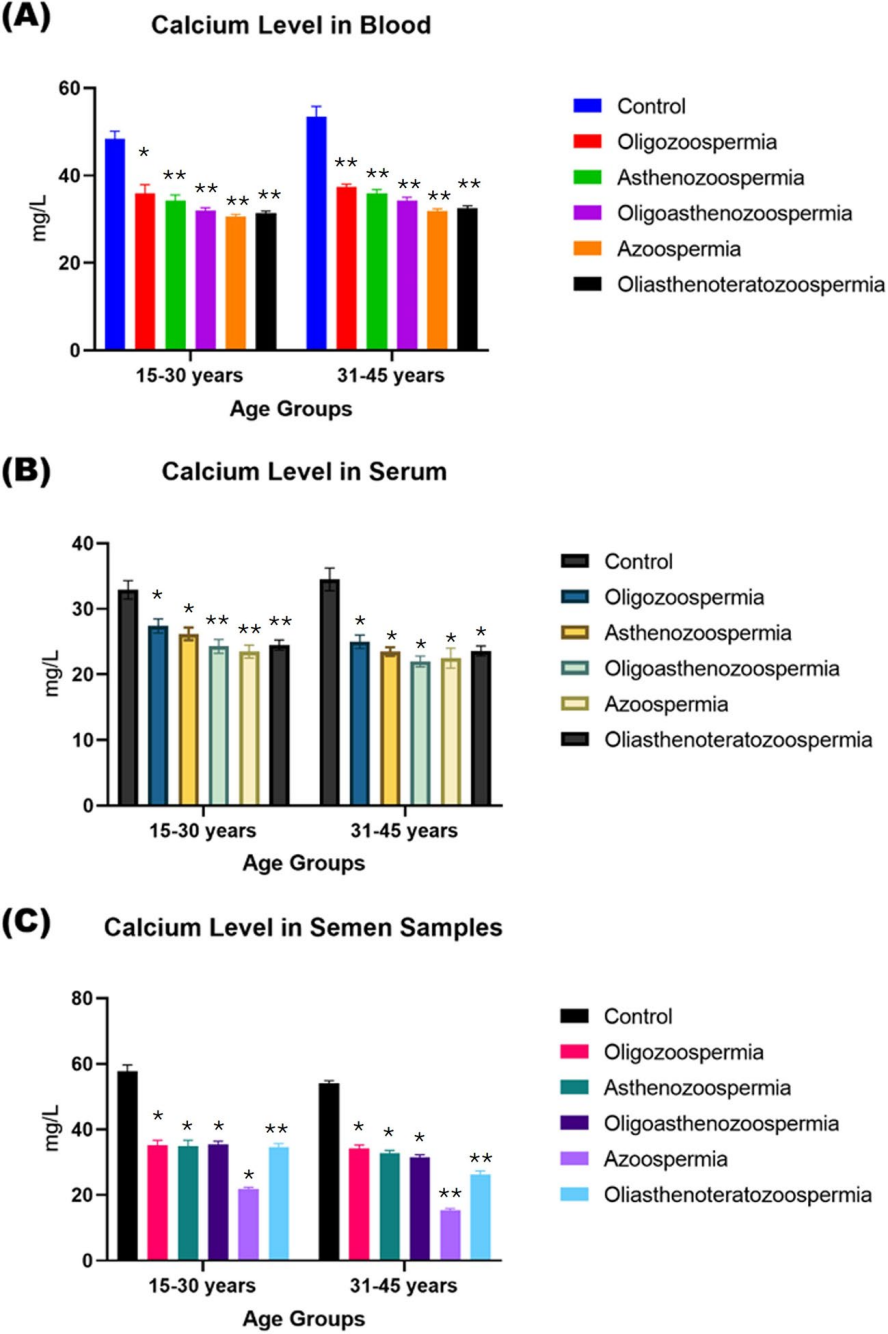
Comparative analysis of Ca concentration (mg/L) across different biological samples—blood (**a**), serum (**b**), and semen (**c**) in normozoospermia control, and patients diagnosed with various subtypes of male infertility. The comparisons are stratified based on age cohorts, namely 15–30 years and 31–45 years. The *p*-value indicates the statistical significance, with values of NS = non-significant (≥ 0.05), * ≤ 0.005, ** ≤ 0.001, *** ≤ 0.01, and **** ≥ 0.01

The levels of Ca in the blood, serum and seminal plasma samples of male subjects aged 15- 30 years, and 31–45 years were found to be 95% confidence interval [95% C.I OR] of [46.2- 48.7, 48.0- 49.9, 51.5- 53.6, 52.4- 54.5] mg/l, [31.5- 33.0, 32.5- 33.4, 33.6- 36.0, 33.5- 35.0] mg/l and [55.0- 58.3, 57.5- 59.5, 55.7- 57.5, 51.5- 53.3] mg/l, respectively. However, the Ca concentrations in blood, serum and seminal plasma samples of all types of infertility patients of both age groups were found to be lower as follows: OZS [34.5- 37.2, 34.6- 38.0, 36.5- 38.5, 38.5- 40.5] mg/l, [(26.4- 28.6, 25.7- 27.3, 24.0- 25.7, 23.0- 25.0] mg/l and [(34.3- 36.4, 35.0- 37.4, 33.5- 36.0, 27.2- 28.5], AZS [(33.0- 35.5, 34.0- 36.7, 35.0- 36.5, 36.0- 37.6] mg/l, (25.5- 26.9, 24.2- 26.5, 23.0- 24.5, 20.5- 22.3] mg/l and [33.5- 36.5, 33.5- 35.9, 32.0- 33.5, 25.0- 26.3 mg/l], OA [31.5- 32.7, 33.0- 34.8, 33.5- 35.2, 35.0- 36.5] mg/l, [23.5- 25.2, 23.5- 25.0, 21.0- 23.9, 20.0- 21.6] mg/l and [35.0- 36.5, 34.0- 36.0, 31.0- 32.5, 23.5- 23.7 mg/l], OAT [30.5- 32.2, 31.5- 32.5, 32.0- 33.5, 31.0- 31.9] mg/l, [24.0- 26.3, 23.5- 24.5, 23.0- 24.4, 20.5- 21.5] mg/l and [33.5- 35.6, 31.0- 34.0, 25.5- 27.2, 18.5- 20.3 mg/l], AZ [30.0- 31.5, 30.2- 31.3, 31.3- 32.5, 29.7- 31.0] mg/l, [22.7- 24.2, 22.5- 23.9, 21.5- 23.5, 18.0- 18.9] mg/l and [21.5- 22.4, 17.5- 18.5, 14.7- 16.0, 12.2- 13.0 mg/l] (Fig. 1).

**Fig. 2:**
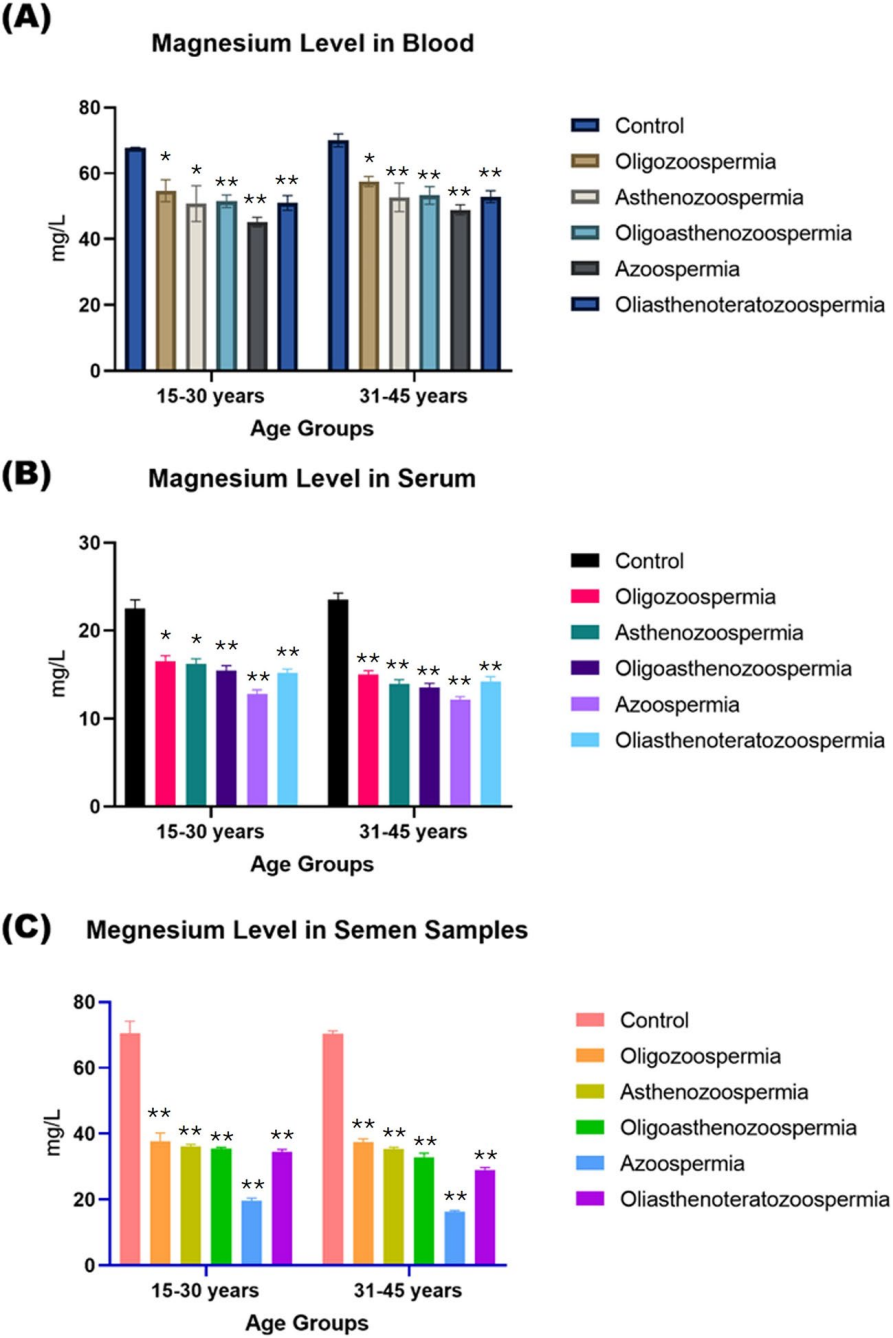
The concentration of Mg (mg/L) in biological samples. Blood (**a**), Serum (**b**), and Semen sample (**c**) among individuals representing normozoospermia control and patients with different phenotype of male infertility. Data comparisons are delineated by age groups, specifically 15–30 years and 31–45 years. The *p*-value indicates the statistical significance, with values of NS = non-significant (≥ 0.05), * ≤ 0.005, ** ≤ 0.001, *** ≤ 0.01, and **** ≥ 0.01

The Mg levels in blood, serum and seminal plasma samples of male adult subjects of both age groups, (15- 30), (25- 34), (35- 44) and (45- 54) years, were found at 95% [CI] as [64.5–68.3, 67.0–70.5, 68.5–70.7, 69.5–72.5] mg/l, [21.5- 23.0, 22.3- 23.5, 23.5- 25.0, 21.3- 22.5] mg/l and [67.5- 69.5, 72.0- 74.5, 71.5- 74.5, 68.0- 69.7] mg/l], respectively. However, the Mg concentrations in blood, serum and seminal plasma samples of all types of infertility patients of both age groups were found to be lower as follows: OZS [53.0—56.5, 55.5- 57.5, 56.2- 58.5, 57.5- 59.3] mg/l, [16.0- 17.2, 15.9—17.0, 14.5- 15.5, 13.5- 14.5] mg/l and [36.2- 39.4, 37.3- 39.5, 37.2- 38.5, 33.2- 34.6], AZS [47.0- 54.3, 49.6- 53.5, 50.2- 55.0, 49.5- 56.3] mg/l, [15.6- 16.3, 14.4- 15.3, 13.5- 14.5, 12.0- 13.0] mg/l and [35.5- 36.7, 36.2- 37.3, 34.7- 36.5, 32.0- 33.3], OA [50.4- 52.7, 51.5- 54.3, 52.0- 54.5, 51.6- 54.2] mg/l, [14.5- 15.9, 14.2- 15.0, 13.0- 14.1, 11.5- 12.3] mg/l and [34.0- 36.9, 35.0- 36.0, 32.0- 33.5, 28.0- 30.0 mg/l], OAT [50.0- 52.5, 50.3- 53.0, 51.5- 53.9, 50.0- 53.5] mg/l, [14.8- 15.7, 13.3- 14.0, 13.5- 14.3, 12.0- 13.2] mg/l and [34.0- 36.3, 32.5- 34.0, 28.3- 29.9, 22.5- 23.0] mg/l, azoospermia [44.2- 46.0, 45.5- 47.5, 48.0- 50.0, 45.3- 46.7] mg/l, [12.3- 13.0, 12.0- 12.5, 11.8- 12.3, 10.5- 11.4] mg/l and [19.0- 19.7, 16.8- 17.7, 15.6- 16.2, 12.0- 12.7] mg/l (Fig. 2).

**Fig. 3:**
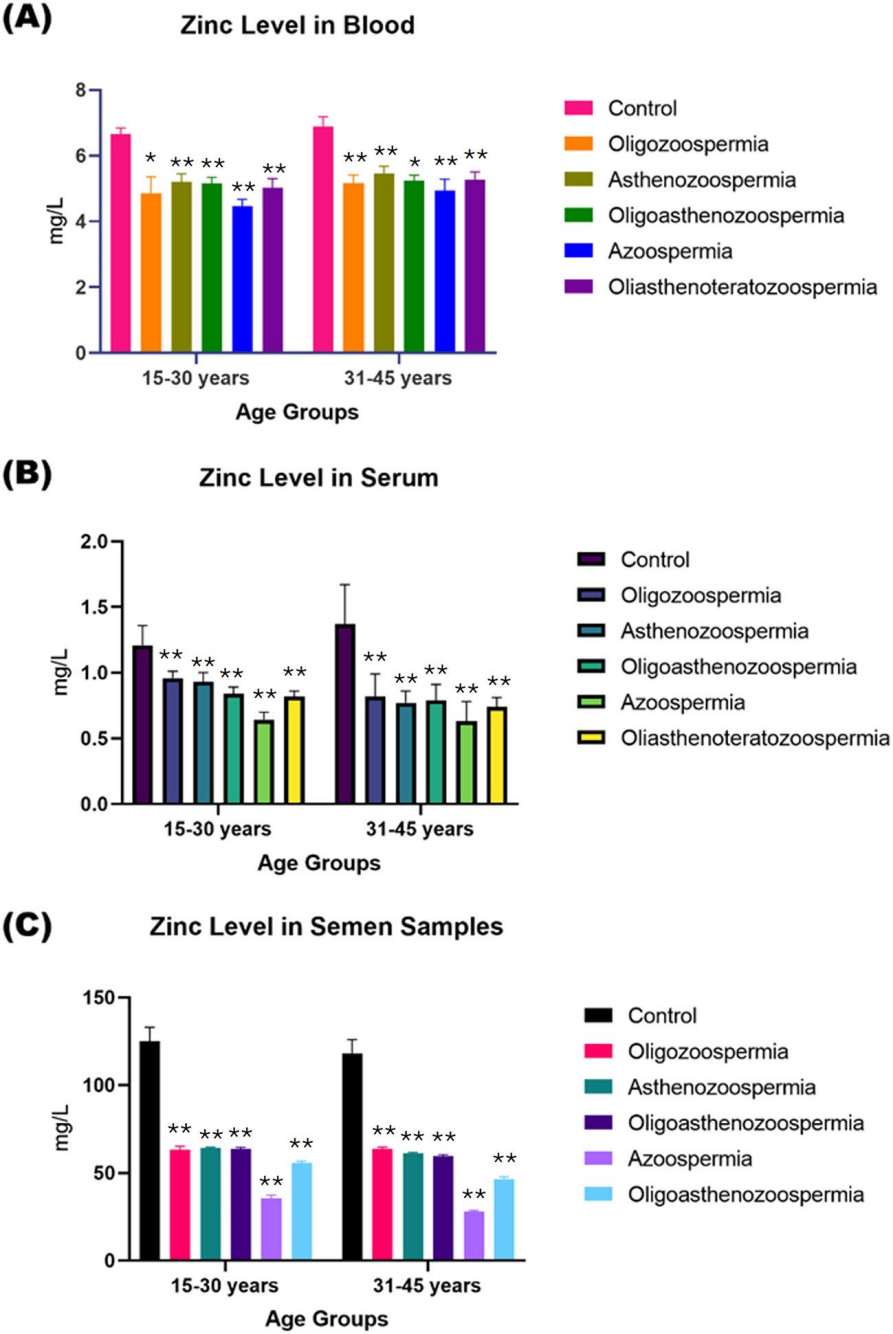
Comparison of Zn concentrations (mg/L) in different biological samples, including blood (**a**), serum (**b**), and semen (**c**), in normozoospermic control patients and patients with different male infertility phenotypes. The data is presented in an age-based format, with subjects divided into those aged 15 to 30 years and 31 to 45 years old. The *p*-value indicates the statistical significance, with values of NS = non-significant (≥ 0.05), * ≤ 0.005, ** ≤ 0.001, *** ≤ 0.01, and **** ≥ 0.01

The Zn levels in blood, serum and seminal plasma samples of male adult subjects of all age groups, (15- 30), (25- 34), (35- 44) and (45- 54) years, were found at 95% [CI] as [6.45–6.72, 6.65–6.80, 6.62–7.03, 6.74–7.02] mg/l, [1.07–1.22, 1.12–1.34, 1.20–1.60, 1.15–1.37] mg/l and [115–122, 120–127, 114–123, 106–115] mg/l], respectively. Whereas the Zn concentrations in blood serum and seminal plasma samples of all types of infertility patients of both age groups were found to be lower as: OA [4.55–5.10, 4.72–5.09, 5.00–5.25, 5.05–5.42] mg/l, [0.90–0.97, 0.91–0.95, 0.71–0.87, 0.65–0.79] mg/l and [62.0–64.5, 63.5–65.0, 63.0–64.5, 54.2–57.3 mg/l], AZS [5.05–5.30, 5.28–5.42, 5.30–5.52, 5.36–5.80] mg/l, [0.86–0.94, 0.75–0.85, 0.70–0.85, 0.55–0.67] mg/l and [63.3–64.2, 63.2–64.2, 60.3–61.7, 51.2–52.0] mg/l, OA [5.00–5.26, 5.15–5.42, 5.16–5.35, 5.12–5.57] mg/l, [0.80–0.89, 0.70–0.81, 0.72–0.84, 0.50–0.65] mg/l and [63.0–64.5, 62.2–63.5, 58.7–60.3, 42.3–43.9], OAT [4.85–5.18, 5.04–5.28, 5.10–5.42, 5.00–5.39] mg/l, [0.78–0.85, 0.60–0.75, 0.70–0.79, 0.57–0.74] mg/l and [55.0–56.5, 51.2–53.9, 45.0–47.0, 42.7–43.2] mg/l, AZ [4.33–4.62, 4.50–4.77, 4.72–5.27,4.10–4.40] mg/l, [0.60–0.69, 0.55–0.64, 0.56–0.72, 0.42–0.60] mg/l and [33.2–36.9, 32.2–33.7, 27.0–28.5, 22.5–23.3] mg/l (Fig. 3).

**Fig. 4:**
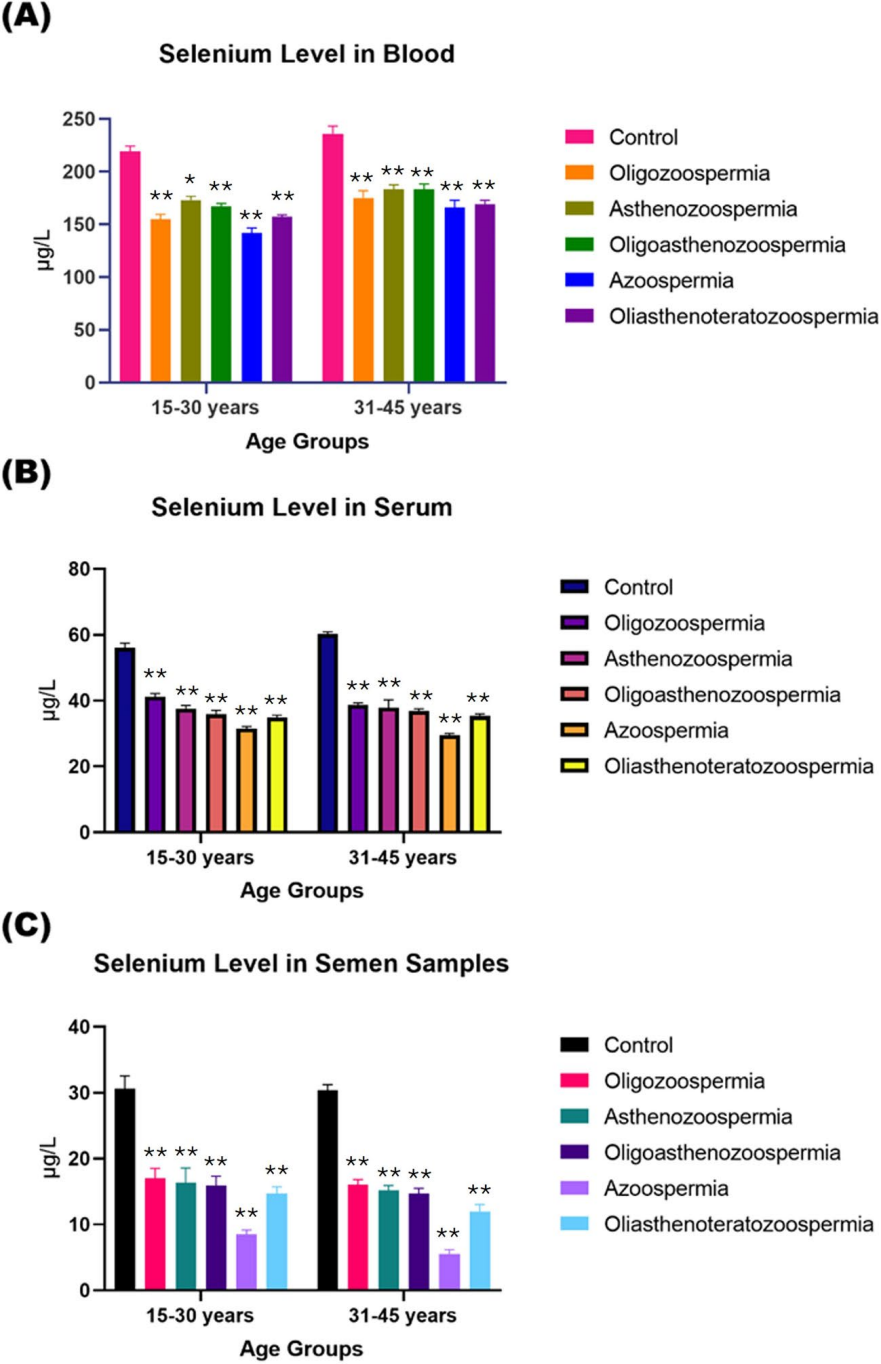
Concentration of Se (µg/L) across different biological matrices—blood (**a**), serum (**b**), and semen (**c**) in individuals representing normozoospermia and different subtypes of male infertility. Data comparisons are delineated by age groups, specifically 15–30 years and 31–45 years. The *p*-value indicates the statistical significance, with values of NS = non-significant (≥ 0.05), * ≤ 0.005, ** ≤ 0.001, *** ≤ 0.01, and **** ≥ 0.01

The Se levels in blood, serum and seminal plasma samples of male adult subjects of four age groups, (15- 30), (25- 34), (35- 44) and (45- 54) years, were found at 95% [CI] as [205–217, 220–235, 230–239, 230–245] µg/l, [54.0–55.5, 56.2–58.5, 61.0–62.0, 57.4–58.9] µg/l and [28.4–30.9, 31.5–33.8, 31.0–32.5, 28.0–28.5] µg/l, respectively. Whereas the Se concentrations in blood, serum and seminal plasma samples of all types of infertility patients of all age groups were found to be lower as: OZS [150–162, 160–176, 170–180, 175–185] µg/l, [40.2–41.9, 40.4–42.5, 38.5–39.5, 35.4–38.0] µg/l and [16.0–17.9, 16.4–18.0, 15.3–16.7, 11.6–12.5] µg/l, AZS [170–175, 175–185, 180–185, 180–187] µg/l, [36.5–38.5, 37.2–39.5, 36.5–39.5, 35.0–36.9] µg/l and [15.2–17.7, 15.6–17.6, 14.6–15.5, 11.0–11.6] µg/l, OA [162–172, 170–179, 175–189, 185–197] µg/l, [35.2–36.7, 35.5–36.9, 36.2–37.5, 34.0–35.4] µg/l and [15.0–16.5, 15.0–16.0, 14.2–15.5, 9.50–10.4] µg/l, OAT [152–163, 158–165, 165–175, 170–175] µg/l, [34.0–35.5, 34.5–35.5, 35.0–36.0, 32.5–33.5] µg/l and [14.0–15.5, 12.0–13.0, 11.0–12.5, 7.20–8.05] µg/l, AZ [136–145, 147–155, 160–170, 145–155] µg/l, [31.0–32.0, 31.5–32.0, 28.5–30.0, 27.5–28.7] µg/l and [8.10–8.75, 6.60–7.35, 5.15–5.75, 3.65–4.35] µg/l, respectively (Fig. 4).

**Fig. 5:**
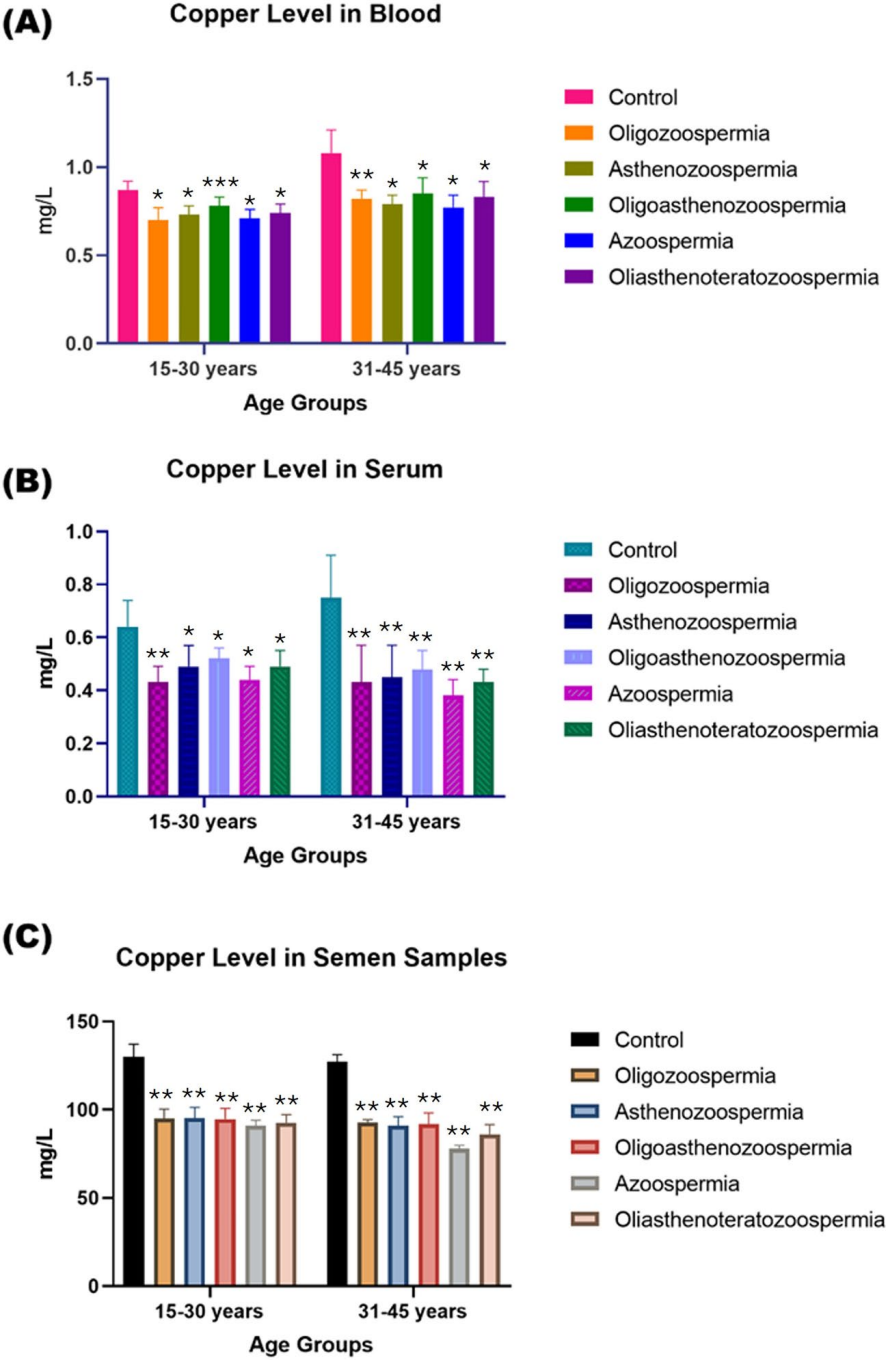
A comparative analysis of Cu concentrations (mg/L) in blood (**a**), serum (**b**), and semen (**c**) in normozoospermia controls and patients with subtypes of male infertility. The data is divided by age groups ranging from 15–30 years and 31–45 years. The *p*-value indicates the statistical significance, with values of NS = non-significant (≥ 0.05), * ≤ 0.005, ** ≤ 0.001, *** ≤ 0.01, and **** ≥ 0.01

**Fig. 6:**
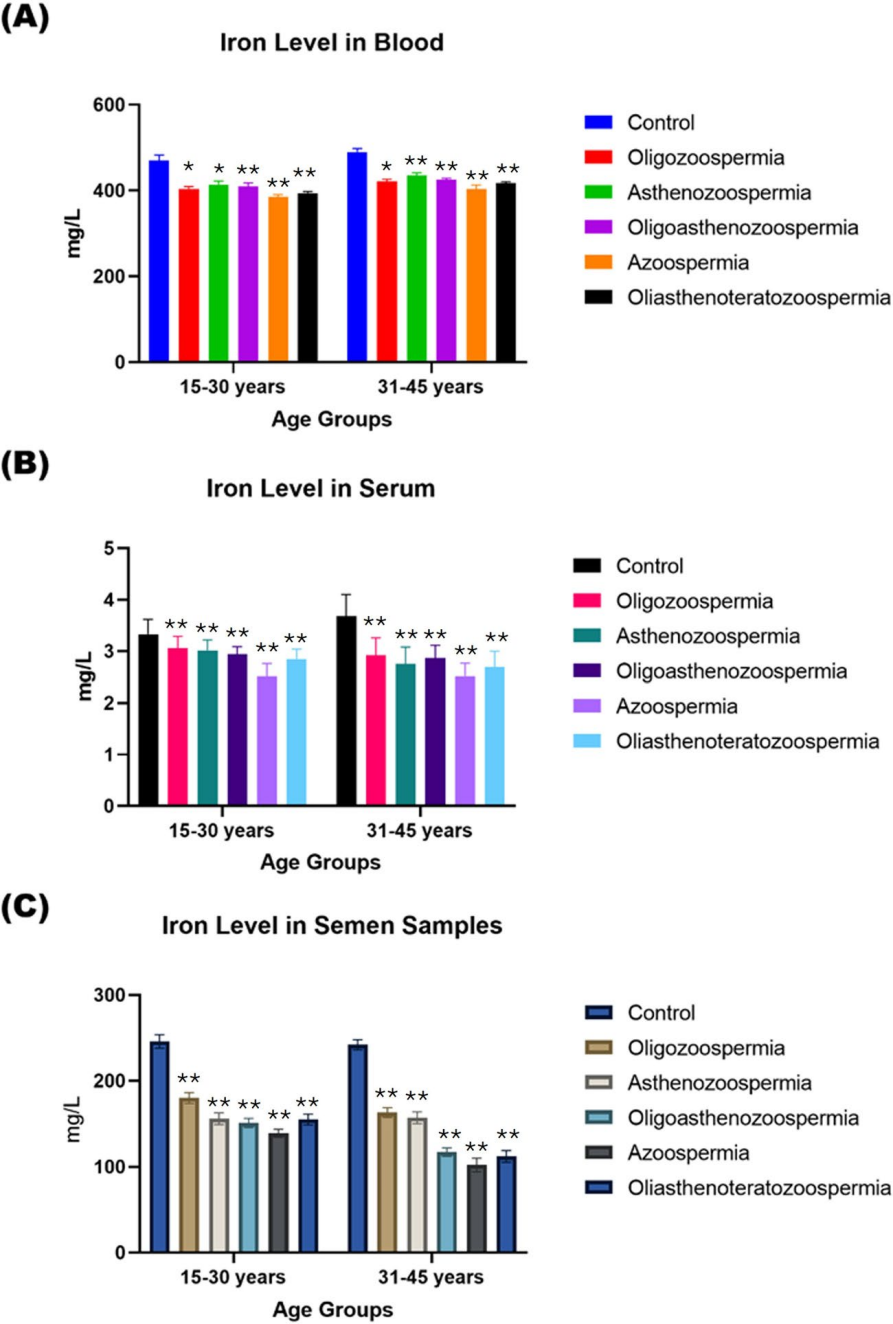
The concentration of Fe (mg/L) in biological samples. Blood (**a**), Serum (**b**), and Semen samples (**c**) among individuals with normozoospermia and diverse forms of male infertility. The examination is stratified based on age groups, encompassing individuals aged 15–30 years and 31–45 years. The *p*-value indicates the statistical significance, with values of NS = non-significant (≥ 0.05), * ≤ 0.005, ** ≤ 0.001, *** ≤ 0.01, and **** ≥ 0.01

The Cu and Fe levels in blood, serum, and seminal plasma samples of adult male subjects of all age groups were found to be higher compared to all subtypes of infertility patients of both age groups (Figs. 5 and 6).

The unpaired Student’s t-test was used to compare the values of electrolytes and essential trace elements levels in biological samples of male, adult, and infertility patients with different degrees of freedom and variable probabilities. At 95 percent confidence intervals, our estimated *t-*value exceeds the *‘t’* critical value, indicating that the difference between the mean values of all selected elements in biological samples from male adult and infertility patients showed significant differences (*p* < 0.01).

## Discussion

In the current study, the concentrations of two electrolytes (Mg and Ca) and four essential trace element concentrations (Cu, Zn, Se, and Fe) were investigated in the serum, blood, and seminal fluid of adult males, and five subtypes of infertile patients in all four age groups. By contributing to the important enzymatic activities required for maintain sperm physiology, these essential trace elements can improve sperm quality in adult males. However, these components may alter sperm function by inducing oxidative stress. These trace elements can improve the sperm quality in adult males by participating in the crucial enzymatic processes necessary to maintain normal sperm physiology. However, these compounds may alter sperm function by inducing oxidative stress.

These findings show that serum samples from experimental patients have lower Ca levels than those from controls. Ca is required for sperm motility, hyperactivity, capacitation, and acrosome reaction in the male reproductive tract [10]. Ca deficiency is associated with male infertility in several ways, including failure of spermatogenesis, impaired steroidogenesis, failure of spermatogenesis, and failure of chemotaxis [31]. High Ca concentration is associated with high of testosterone production in Leydig cells, while the Ca chelator limits the growth of steroids [32].

Magnesium (Mg) is a key electrolyte involved in the spermatogenic phase and sperm motility [16]. Therefore, lower Mg concentrations in infertile patients may indicate that their prostate glands are not unhealthy. In the case of prostate infection, magnesium levels in the body decrease significantly. The increase in nitric oxide levels is associated with low magnesium levels, resulting in premature ejaculation [16]. Omu et al. found a correlation between magnesium levels and premature ejaculation. In the female reproductive system, Mg is also essential for sperm motility [16]. The Mg2 + dependent ATPase releases the energy for sperm from ATP. Several studies have reported higher levels of Mg in human semen, testis, seminal vesicles, and prostate glands. Mg concentrations are positively related to Ca concentrations [16].

The results showed that the concentrations of Zn in blood and serum samples from infertility patients were significantly lower than those in the adult group (*p* < 0.01). Zinc deficiency is associated with decreased sexual maturity, hypogonadism, gonadal dysfunction, testicular weight loss, damage to Leydig cells and seminiferous tubules, and testicular atrophy [31, 33]. Male sex hormones, such as testosterone, require zinc for production, storage, and secretion [31]. Testosterone is a hormone produced by Leydig cells that affects sperm production.

Some studies, have found a correlation between seminal plasma Zn concentration, testicular steroidogenesis, and serum-free testosterone [31]. Zn deficiency is associated with failure of steroidogenesis, decreased testosterone and progesterone levels, and increased luteinizing hormones and follicle stimulate hormone [22]. Zn deficiency causes Leydig cells to death, decreased testosterone levels, to decline, and failure of spermatogenesis[22]. Zn is required for DNA replication, transcription, packaging, protein production, cell differentiation, and proliferation, which contributes to sperm production [34]. The current study found that Zn deficiency causes an increase in reactive oxygen species (ROS) and oxidative stress, which decrease sperm quality and causes male infertility [34]. Zn deficiency reduces antioxidant defenses and increases inflammatory susceptibility in sperm [34–38].

Fe is involved in the reduction of oxidation reactions and is present in many enzymes and metal–protein molecules [36]. Marzec-Wroblewska et al. [36] found that, contrary to current results, higher Fe levels, had a harmful effect on sperm morphology.

Cu is necessary for the activity of several metal enzymes involved in energy and antioxidant metabolism (such as Cu/Zn-SOD, C-chromosome oxidase, and tyrosine). It also protects sperm cells from oxidative damage through its role in the redox system. However, Cu has a negative impact on human sperm if consumed in large amounts [36].

Recent studies have shown that high Cu concentrations in seminal plasma are associated with sperm DNA damage [38]. Thus, our data suggest that Cu is essential for the function of healthy spermatozoa, but it may cause toxicity and harmful effects on sperm quality at higher doses.

Selenium (Se) is another essential trace element that is needed to maintain male fertility. It is also an antioxidant component of glutathione peroxidase. According to [39], dietary Se deficiency induces oxidative stress and has a deleterious effect on spermatogenesis. Two essential enzymes involved in sperm production are selenoprotein and glutathione-peroxidase phospholipid hydrogen peroxidase (PHGPx). The most common selenoprotein synthesized by testicular germ cells is PHGPx, which functions as a link between selenium, sperm quality, and male fertility [39]. Se can have a beneficial effect on Leydig cells, causing a change in testosterone secretion. Consequently, low basal Se levels are associated with poor sperm quality and an increased risk of male infertility. Se is strongly correlated with sperm count, sperm motility, proper sperm morphology, and vitality of seminal plasma [31]. Some researchers have studied the effects of Se on sperm quality and fertility in men with abnormal sperm parameters [31]. Morbat et al. reported that supplementing infertile men with Se (50 µg/day for 3 months) dramatically boosts sperm count, motility, viability, and normal morphology, as well as ejaculate volume [40].

There was a correlation (*r*) between the Ca, Mg, Se, and Zn contents in serum, blood, and seminal plasma samples and biochemical and chemical parameters (hemoglobin, serum testosterone, total erythrocyte count, sperm count, percentage of motile sperm, and rapid linear progression) in healthy adults and patients with various types of infertility in the age groups 15–30 and 31–45 years. The Ca, Mg, Se, and Zn contents in these biological samples correlated (*r*) with biochemical and clinical parameters in healthy adult participants (*r* = 0.62–0.75), while an association (*r* = 0.06–0.32) was observed in biological samples from infertile patients.

Oxidative stress is believed to be a primary component of infertility. When the body's reactive oxygen species (ROS) and antioxidants are imbalanced, oxidative stress results in sperm damage, deformity, and male infertility. ROS are free radicals that are involved in several physiological processes in sperm, including capacitation, hyperactivation, and sperm-oocyte fusion [40, 41]. In contrast, prolonged exposure to certain heavy metals or their compounds can damage nucleic acids, cause mutations, mimic hormones, disrupt endocrine and reproductive systems, and ultimately result in cancer.

## Strength and Limitations

Our study has demonstrated the promising potential of key trace elements and antioxidant supplements in enhancing sperm quality, which could have a substantial negative impact on human reproductive health and contribute to male infertility. Consequently, our research offers valuable insights that may inform diagnostic and therapeutic approaches for male reproductive health. Although the study area was limited to one city, we were able to recruit a substantial number of participants, which constitutes the only limitation of our research.

## Conclusions

This is a significant study because it is the first in the region to examine the levels of electrolytes and essential trace element in biological samples (serum, blood, and seminal plasma) of healthy adult male subjects and infertility patients on the basis of spermiogram findings of both age groups (15–30) and (31–45). Lower levels of electrolytes (Ca and Mg) and essential trace elements in seminal plasma, especially Zn and Se, are directly proportional to the increased production of reactive oxygen species, which may be associated with low sperm motility and male infertility. Therefore, it is likely that a loss of the oxidant-reductant equilibrium or an oxidant-reductant imbalance caused by decreasing levels of important trace elements in seminal plasma could impair male fertility. These findings highlight the value of conducting large-scale, controlled, randomized research to determine the effectiveness of key trace elements and antioxidant supplements on sperm quality.

## Supplementary Information

Below is the link to the electronic supplementary material.

Supplementary file1 (DOCX 53 KB)

Supplementary file2 (DOCX 503 KB)

## Data Availability

No datasets were generated or analysed during the current study.
