# Supplementary material for: Determination of Electrolytes and Trace Elements in Biological Samples from Patients with Altered Semen Parameters: a Correlational Analysis
Source: Biol Trace Elem Res. 2024 Jun 26;203(3):1383–94. doi: 10.1007/s12011-024-04281-7 (PMC11872761; doi:10.1007/s12011-024-04281-7)
Supplement: Supplementary file 1 — Supplementary file1 (DOCX 53 KB) [file 12011_2024_4281_MOESM1_ESM.docx]

**Supplementary Table S1. Elemental concentrations in certified samples (CRM) by microwave digestion method (MWD)**

| **Elements** | **Certified values** | **MWD** | **T _value_ ^a^** | **% Recovery^b^** |
| --- | --- | --- | --- | --- |
| **CRM of whole serum (mg /L)** | | | | |
| **Iron** | 1.30±0.3 | 1.286±0.087  (7.65) | 0.824 | 98.9 |
| **Copper** | 1.2±0.2 | 1.194±0.074  (6.20) | 0.855 | 99.5 |
| **Zinc** | 0.97±0.01 | 0.965±0.019  (1.97) | 0.376 | 99.5 |
| **Selenium** | ------- | ------ | ------ | ---------- |
| **Calcium** | 58 ±1.9 | 57.4 ±0.95  (1.65) | 0.00415 | 98.9 |
| **Magnesium** | 8.1 ±0.36 | 8.05 ±0.087  (1.08) | 0.00362 | 99.4 |
| **CRM of whole blood (mg /L)** | | | | |
| **Iron** | 14.2±3.24 | 14.15±0.76 (5.37) | 0.935 | 99.6 |
| **Copper** | 13.9±2.7 | 13.85±0.97  (7.00) | 0.910 | 99.6 |
| **Zinc** | 2.27±0.06 | 2.24±0.05  (2.23) | 0.304 | 98.7 |
| **Selenium** | 116±23.5 | 115.2±2.56  (2.22) | 0.236 | 99.3 |
| **Calcium** | 14.7 ±0.3 | 14.62 ±1.15  (7.86) | 0.865 | 99.4 |
| **Magnesium** | 17.0 ±0.3 | 16.93 ±1.56  (9.21) | 0.935 | 99.6 |

**Key:- Values in ( ) are %RSD,**

**^Paired t-test between Certified Value and MWD DF = 5,
T (critical) at 95 % CI = 2.57, p < 0.50,
$ *%* recovery was calculated according to : ([MDM])/([Certified Value]) x 100**

**Supplementary Table S2. Elemental concentrations in biological samples of in adult healthy referent and different types of infertility patients**

| **Referents/ Patients** | **Age**  **years** | **Calcium**  **mg/L** | **Magnesium**  **mg/L** | **Zinc**  **mg/L** | **Selenium µg/L** | **Iron**  **mg/L** | **Copper**  **mg/L** |
| --- | --- | --- | --- | --- | --- | --- | --- |
| **Blood** | | | | | | | |
| **Referent** | **15-30** | 48.4 ±1.75  46.7- 49.7 | 67.7±2.90  64.7-69.5 | 6.65±0.19  6.56-6.75 | 219±5.29  207-223 | 470±12.5  455-478 | 0.87±0.05  0.79-0.92 |
| **Oligozoospermia** |  | 35.9 ±2.05  35.0- 37.0 | 54.7±3.35  52.7- 56.2 | 4.85±0.51  4.59-5.12 | 155±4.52  152-158 | 404±5.32  401-406 | 0.70±0.07  0.67-0.74 |
| **p- value** |  | 0.005 | 0.004 | 0.002 | 0.001 | 0.003 | 0.005 |
| **Asthenozoospermia** |  | 34.2 ±1.35  33.5- 34.9 | 50.8±5.45  47.4- 54.0 | 5.21±0.24  5.09-5.32 | 173±3.55  171-175 | 414±8.34  410-417 | 0.73±0.05  0.70-0.76 |
| **p- value** |  | 0.001 | 0.003 | 0.001 | 0.002 | 0.002 | 0.007 |
| **Oligoasthenozoospermia** |  | 32.0 ±0.62  31.7- 32.4 | 51.5±1.90  50.6- 52.6 | 5.15±0.19  5.06-5.24 | 167±2.82  165-170 | 410±7.65  407-414 | 0.78±0.05  0.76-0.80 |
| **p- value** |  | 0.001 | 0.001 | 0.001 | 0.001 | 0.001 | 0.011 |
| **Oliasthenoteratozoospermia** |  | 31.3 ±0.59  31.0- 31.7 | 51.0±2.25  49.9- 52.3 | 5.03±0.27  4.87-5.15 | 157±1.98  155-160 | 394±3.60  392-396 | 0.74±0.05  0.72-0.76 |
| **p- value** |  | 0.001 | 0.001 | 0.001 | 0.001 | 0.001 | 0.004 |
| **Azoospermia** |  | 30.6 ±052  30.3- 31.0 | 45.2±1.45  44.5- 45.8 | 4.47±0.21  4.36-4.57 | 142±4.52  139-145 | 385±5.19  382-389 | 0.71±0.05  0.68-0.73 |
| **p- value** |  | 0.001 | 0.001 | 0.001 | 0.001 | 0.001 | 0.002 |
| **Referent** | **31-45** | 53.4 ±2.42  51.7- 53.3 | 70.0±1.95  68.6-70.4 | 6.89±0.30  6.65-7.02 | 236±7.30  231-237 | 489±8.82  477-486 | 1.08±0.13  1.00-1.15 |
| **Oligozoospermia** |  | 37.4 ±0.62  36.9- 37.8 | 57.5±1.53  56.7- 58.3 | 5.17±0.25  5.02-5.30 | 175±6.92  172-178 | 421±5.22  417-424 | 0.82±0.05  0.79-0.85 |
| **p- value** |  | 0.001 | 0.002 | 0.001 | 0.001 | 0.003 | 0.001 |
| **Asthenozoospermia** |  | 35.9 ±0.85  35.3- 36.4 | 52.7±4.35  50.5- 55.0 | 5.45±0.23  5.34-5.56 | 183±4.65  180-185 | 435±6.07  432-439 | 0.79±0.05  0.77-0.81 |
| **p- value** |  | 0.001 | 0.001 | 0.001 | 0.001 | 0.001 | 0.002 |
| **Oligoasthenozoospermia** |  | 34.2 ±0.82  33.7- 34.6 | 53.3±2.65  52.0- 54.7 | 5.24±0.17  5.18-5.32 | 183±5.29  179-186 | 425±3.62  422-428 | 0.85±0.09  0.81-0.89 |
| **p- value** |  | 0.001 | 0.001 | 0.002 | 0.001 | 0.001 | 0.005 |
| **Oliasthenoteratozoospermia** |  | 32.5 ±0.59  32.20.001-0.00232.9 | 52.9±1.82  51.9- 53.7 | 5.27±0.24  5.15-5.40 | 169±4.09  167-172 | 417±3.09  415-420 | 0.83±0.09  0.79-0.87 |
| **p- value** |  | 0.001 | 0.001 | 0.001 | 0.001 | 0.001 | 0.003 |
| **Azoospermia** |  | 31.9 ±0.46  31.6- 32.3 | 48.9±1.55  48.2- 49.7 | 4.94±0.35  4.76-5.23 | 166±6.92  163-170 | 403±9.85  397-409 | 0.77±0.07  0.75-0.80 |
| **p- value** |  | 0.001 | 0.001 | 0.001 | 0.001 | 0.001 | 0.002 |
|  |  |  |  | **Serum** |  |  |  |
| **Referent** | **15-30** | 32.9 ±1.40  31.7- 33.0 | 22.5± 0.99  21.7- 22.7 | 1.21±0.15  1.10-1.25 | 56.0±1.49  54.0-55.6 | 3.33±0.29  3.19-3.52 | 0.64±0.10  0.57-0.69 |
| **Oligozoospermia** |  | 27.4 ±1.07  26.9- 28.0 | 16.5±0.65  16.2- 16.8 | 0.96±0.05  0.93-0.99 | 41.2±0.98  40.6-41.7 | 3.05±0.24  2.92-3.16 | 0.43±0.06  0.40-0.47 |
| **p- value** |  | 0.003 | 0.002 | 0.001 | 0.001 | 0.001 | 0.001 |
| **Asthenozoospermia** |  | 26.2 ±0.95  25.7- 26.7 | 16.2±0.59  15.8- 16.5 | 0.93±0.07  0.89-0.96 | 37.5±1.09  36.8-38.2 | 3.02±0.20  2.91-3.13 | 0.49±0.08  0.45-0.54 |
| **p- value** |  | 0.002 | 0.002 | 0.001 | 0.001 | 0.001 | 0.007 |
| **Oligoasthenozoospermia** |  | 24.3 ±1.05  23.8- 24.9 | 15.4±0.62  15.0- 15.6 | 0.84±0.05  0.82-0.87 | 35.9±1.12  35.3-36.5 | 2.94±0.15  2.86-3.03 | 0.52±0.04  0.50-0.55 |
| **p- value** |  | 0.001 | 0.001 | 0.001 | 0.001 | 0.001 | 0.009 |
| **Oliasthenoteratozoospermia** |  | 24.5 ±0.75  24.2- 25.8 | 15.2±0.40  15.0- 15.5 | 0.82±0.04  0.80-0.84 | 34.8±0.75  34.4-35.3 | 2.85±0.19  2.74-2.96 | 0.49±0.06  0.46-0.52 |
| **p- value** |  | 0.001 | 0.001 | 0.001 | 0.001 | 0.001 | 0.007 |
| **Azoospermia** |  | 23.5 ±0.99  23.0- 24.0 | 12.8±0.45  12.5- 13.0 | 0.64±0.06  0.61-0.67 | 31.5±0.64  31.2-31.9 | 2.51±0.25  2.42-2.65 | 0.44±0.05  0.42-0.47 |
| **p- value** |  | 0.001 | 0.001 | 0.001 | 0.001 | 0.001 | 0.002 |
| **Referent** | **31- 45** | 34.5±1.72  33.8- 35.9 | 23.5± 0.75  23.8- 24.7 | 1.37±0.30  1.23-1.59 | 60.2±0.70  61.2-62.0 | 3.68±0.42  3.57-3.93 | 0. 75±0.16  0.70-0.93 |
| **Oligozoospermia** |  | 25.0 ±1.02  24.5- 25.4 | 15.0±0.42  14.7- 15.2 | 0.82±0.17  0.74-0.90 | 38.7±0.63  38.4-39.0 | 2.92±0.34  2.74-3.09 | 0.43±0.14  0.36-0.49 |
| **p- value** |  | 0.002 | 0.001 | 0.001 | 0.001 | 0.001 | 0.001 |
| **Asthenozoospermia** |  | 23.5 ±0.65  23.2- 23.8 | 13.9±0.52  13.6- 14.3 | 0.77±0.09  0.72-0.84 | 37.9±2.36  36.7-39.2 | 2.75±0.33  2.58-2.95 | 0.45±0.12  0.37-0.52 |
| **p- value** |  | 0.002 | 0.001 | 0.001 | 0.001 | 0.001 | 0.001 |
| **Oligoasthenozoospermia** |  | 22.0 ±0.80  21.5- 23.4 | 13.5±0.48  13.2- 13.8 | 0.79±0.12  0.73-0.85 | 36.8±0.70  36.4-37.2 | 2.87±0.25  2.74-2.99 | 0.48±0.07  0.45-0.51 |
| **p- value** |  | 0.002 | 0.001 | 0.001 | 0.001 | 0.001 | 0.001 |
| **Oliasthenoteratozoospermia** |  | 23.6 ±0.75  23.2- 24.0 | 14.2±0.55  13.9- 14.5 | 0.74±0.07  0.71-0.77 | 35.3±0.65  35.0-35.7 | 2.69±0.31  2.35-2.87 | 0.43±0.05  0.40-0.45 |
| **p- value** |  | 0.002 | 0.001 | 0.001 | 0.001 | 0.001 | 0.001 |
| **Azoospermia** |  | 22.5 ±1.52  21.7- 23.4 | 12.2±0.31  12.0- 12.4 | 0.63±0.15  0.57-0.69 | 29.5±0.52  28.9-29.9 | 2.52±0.25  2.39-2.62 | 0.38±0.06  0.35-0.41 |
| **p- value** |  | 0.002 | 0.001 | 0.001 | 0.001 | 0.001 | 0.001 |
|  |  |  | **Seminal plasma** |  |  |  |  |
| **Referent** | **15-30** | 57.8±1.89  55.4- 58.6 | 70.5± 3.65  67.7- 74.0 | 125± 8.06  117-128 | 30.6±1.95  28.7-32.5 | 246± 7.90  239-253 | 130±7.15  125-136 |
| **Oligozoospermia** |  | 35.2±1.50  34.5- 35.9 | 37.6± 2.65  36.3- 39.0 | 63.2±1.96  62.3-64.4 | 17.0±1.52  16.3-17.7 | 180± 6.29  176-184 | 94.9±5.42  92.3-97.2 |
| **p- value** |  | 0.002 | 0.001 | 0.001 | 0.001 | 0.001 | 0.001 |
| **Asthenozoospermia** |  | 34.9±1.77  33.9- 36.0 | 36.2± 0.55  35.9- 36.5 | 64.0±0.72  63.6-64.4 | 16.4±2.19  15.3-17.5 | 156± 6.82  152-160 | 95.3±6.05  92.0-97.9 |
| **p- value** |  | 0.002 | 0.001 | 0.001 | 0.001 | 0.001 | 0.001 |
| **Oligoasthenozoospermia** |  | 35.4±1.05  35.0- 36.2 | 35.4± 0.51  34.2- 36.7 | 63.5±1.05  63.0-64.2 | 15.9±1.40  15.2-16.5 | 151± 5.40  148-154 | 94.8±6.02  91.0-98.2 |
| **p- value** |  | 0.002 | 0.001 | 0.001 | 0.001 | 0.001 | 0.001 |
| **Oliasthenoteratozoospermia** |  | 34.5±1.17  33.8- 35.2 | 34.5± 0.72  34.2- 35.8 | 55.7±0.95  55.2-56.2 | 14.7±1.03  14.2-15.4 | 155± 6.37  151-158 | 92.5±4.67  91.9-95.0 |
| **p- value** |  | 0.002 | 0.001 | 0.001 | 0.001 | 0.001 | 0.001 |
| **Azoospermia** |  | 21.9±0.47  21.6- 22.3 | 19.6± 0.82  19.2- 20.0 | 35.4±1.95  33.5-36.5 | 8.47±0.72  8.14-8.75 | 139± 4.52  154-160 | 90.9±3.12  88.2-92.4 |
| **p- value** |  | 0.001 | 0.001 | 0.001 | 0.001 | 0.001 | 0.001 |
| **Referent** | **31-45** | 54.0±0.86  56.2- 55.0 | 70.3± 0.95  69.7- 71.9 | 118± 7.95  114-121 | 30.4±0.85  29.9-31.5 | 242± 5.95  239-246 | 127±4.22  124-130 |
| **Oligozoospe8rmia** |  | 34.2±1.05  33.7- 35.7 | 37.5± 0.92  37.0- 38.2 | 63.7±1.02  63.2-64.3 | 16.0±0.80  15.6-16.5 | 163± 5.55  160-166 | 92.9±1.52  92.2-93.6 |
| **p- value** |  | 0.002 | 0.001 | 0.001 | 0.001 | 0.001 | 0.001 |
| **Asthenozoospermia** |  | 32.7±0.91  32.2- 33.3 | 35.3± 0.62  34.9- 36.7 | 61.0±0.73  60.6-61.5 | 15.2±0.73  14.8-15.4 | 157± 6.82  153-160 | 90.9±5.19  88.5-93.2 |
| **p- value** |  | 0.002 | 0.001 | 0.001 | 0.001 | 0.001 | 0.001 |
| **Oligoasthenozoospermia** |  | 31.6±0.67  31.2- 32.0 | 32.9± 1.20  32.3- 33.4 | 59.5±0.96  59.0-59.9 | 14.7±0.78  14.3-15.2 | 117± 4.92  114-120 | 91.9±6.30  88.7-97.0 |
| **p- value** |  | 0.002 | 0.001 | 0.001 | 0.001 | 0.001 | 0.001 |
| **Oliasthenoteratozoospermia** |  | 26.3±1.08  25.7- 26.9 | 29.0± 0.79  28.6- 29.4 | 46.3±1.52  45.4-47.3 | 11.9±1.15  11.3-12.5 | 112± 6.97  108-116 | 85.9±5.69  83.0-88.7 |
| **p- value** |  | 0.001 | 0.001 | 0.001 | 0.001 | 0.001 | 0.001 |
| **Azoospermia** |  | 15.3±0.59  15.0- 15.7 | 16.2± 0.49  15.9- 16.4 | 27.9±0.70  27.5-28.3 | 5.48±0.69  5.15-5.72 | 102± 7.85  98.4-106 | 77.9±1.96  76.9-79.0 |
| **p- value** |  | 0.001 | 0.001 | 0.001 | 0.001 | 0.001 | 0.001 |
