# Supplementary material for: Determination of Electrolytes and Trace Elements in Biological Samples from Patients with Altered Semen Parameters: a Correlational Analysis
Source: Biol Trace Elem Res. 2024 Jun 26;203(3):1383–94. doi: 10.1007/s12011-024-04281-7 (PMC11872761; doi:10.1007/s12011-024-04281-7)
Supplement: Supplementary file 2 — Supplementary file2 (DOCX 503 KB) [file 12011_2024_4281_MOESM2_ESM.docx]

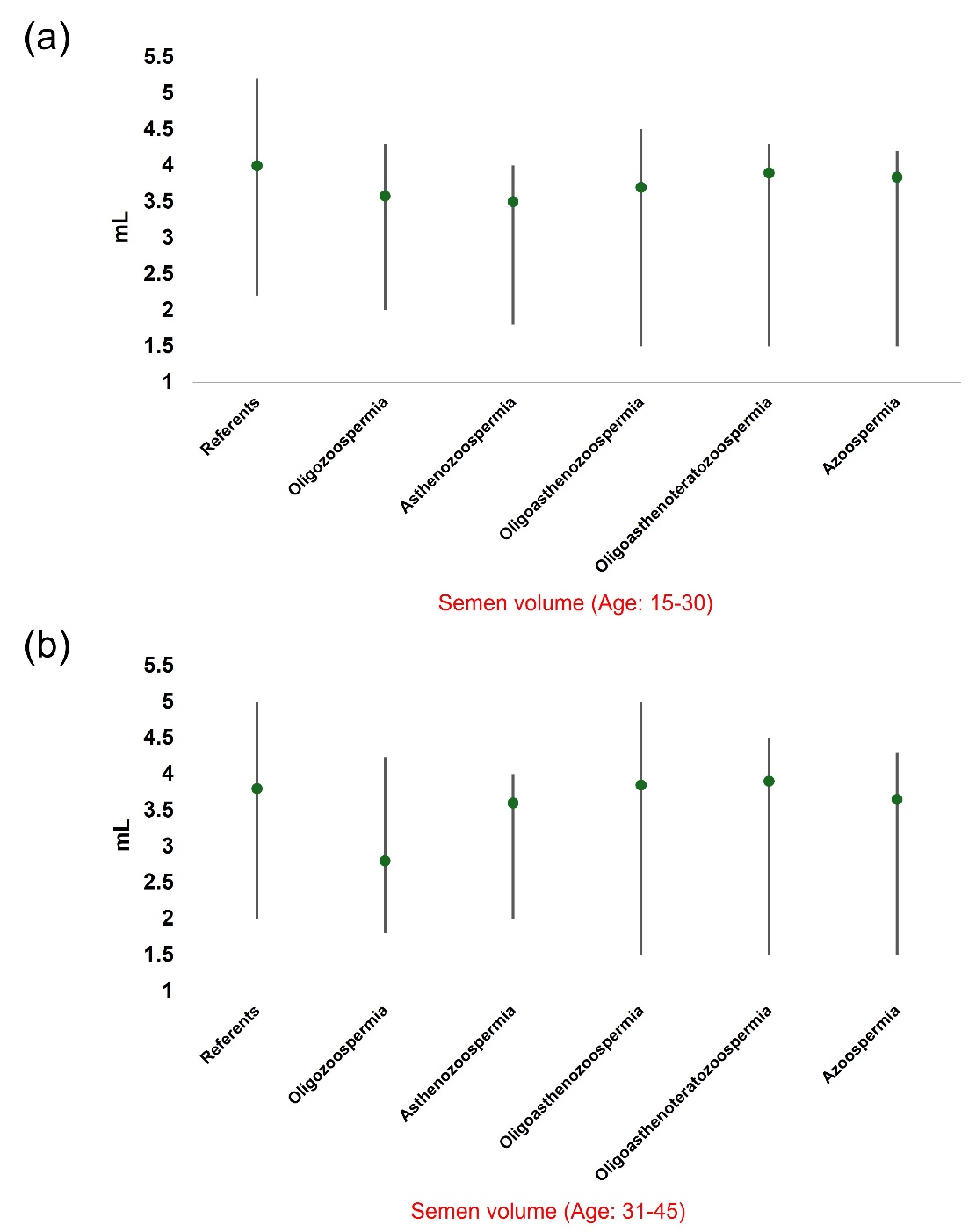


Supplementary Figure S1. Comparison of semen volume across various sperm disorders and the normal range for two different age groups: (a) 15-30 years and (b) 31-45 years.


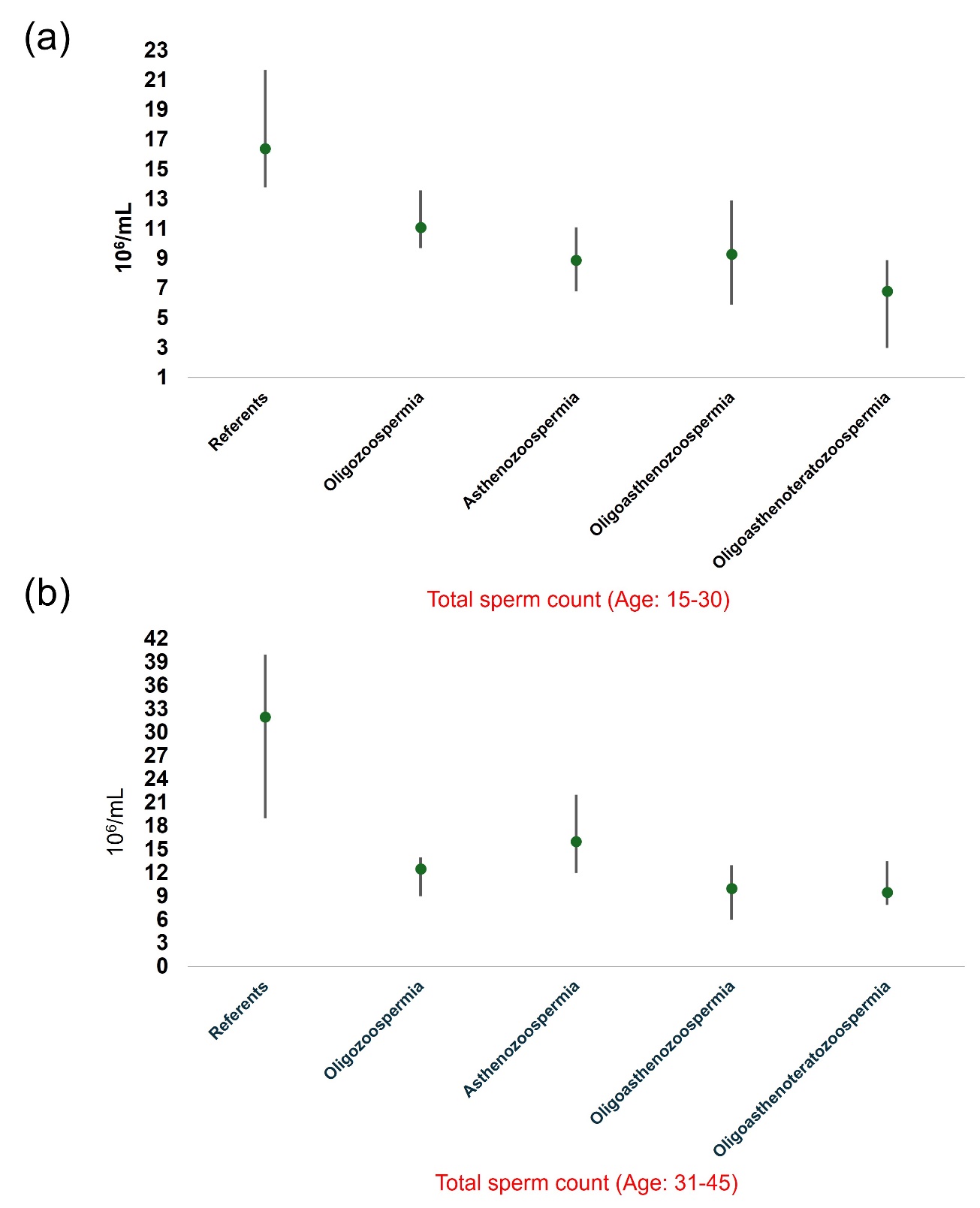


Supplementary Figure S2. Comparison of semen volume across various sperm disorders and the control range for two different age groups: (a) 15-30 years and (b) 31-45 years.


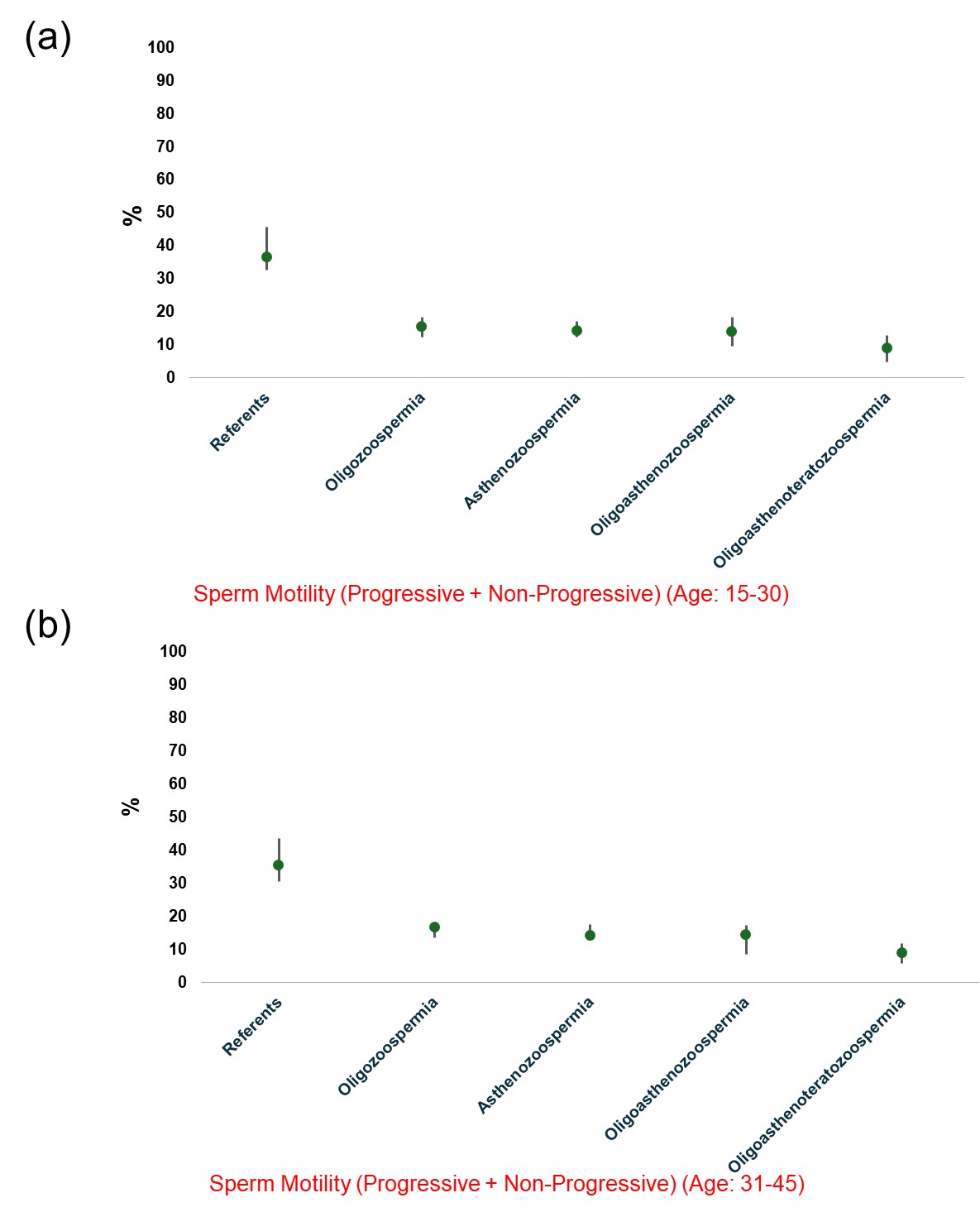


Supplementary Figure S3. The motility of spermatozoa (progressive + non-progressive motility) across various sperm disorders. The data is stratified into two distinct age groups: (a) 15-30 years and (b) 31-45 years. Motility is presented as percentage values. The median value in each category is represented by a green dot.


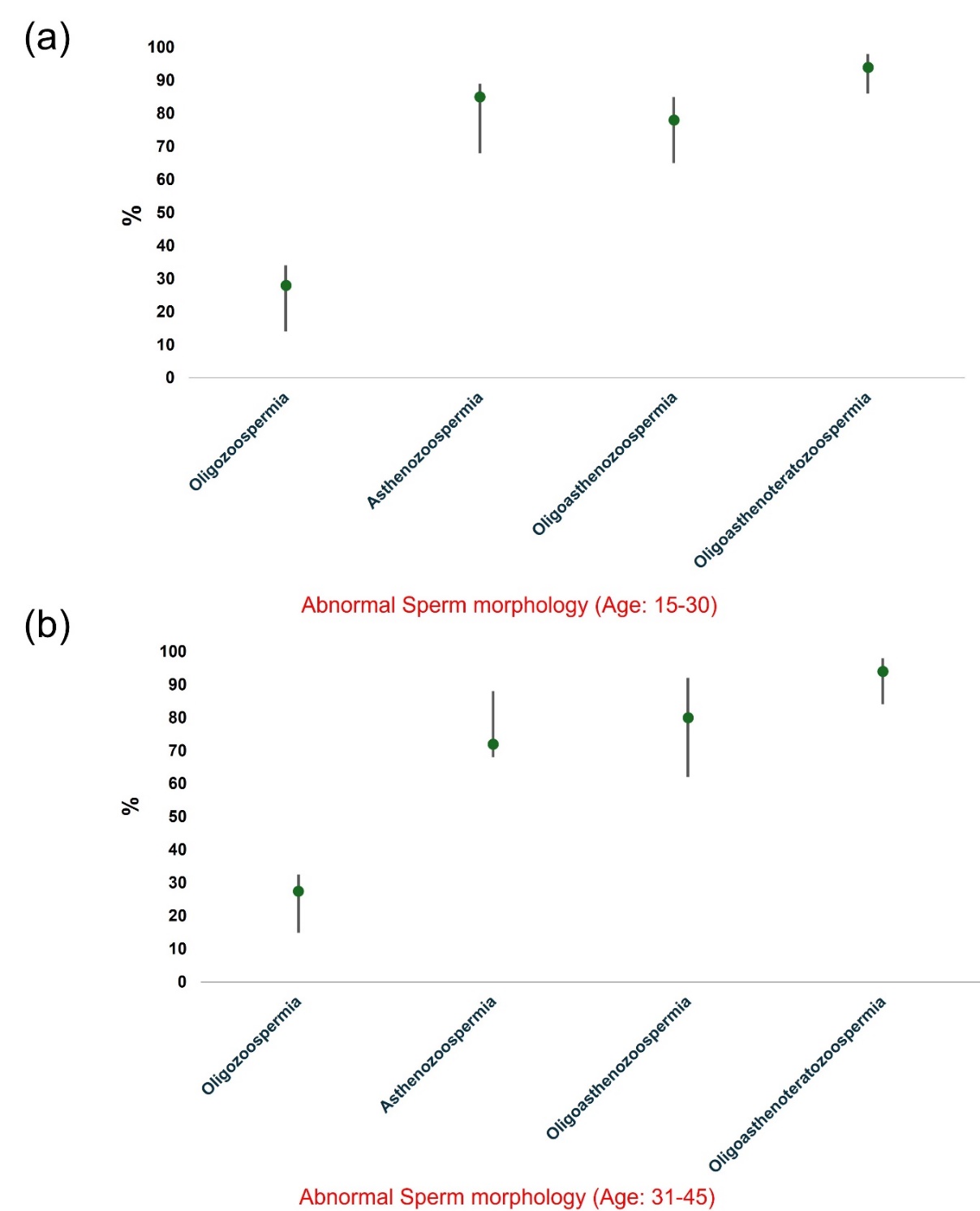


Supplementary Figure S4. Abnormal sperm morphology in various sperm disorders for two different age groups: (a) 15-30 years and (b) 31-45 years, presented as percentage values. The green dot indicates the median value.
